# A theory of moving form perception: Synergy between masking, perceptual grouping, and motion computation in retinotopic and non-retinotopic representations

**DOI:** 10.2478/v10053-008-0015-2

**Published:** 2008-07-15

**Authors:** Haluk Öğmen

**Affiliations:** Department of Electrical & Computer Engineering, Center for Neuro-Engineering & Cognitive Science, University of Houston, Houston, TX 77204-4005 USA

**Keywords:** moving form perception, dynamic form perception, visual masking, perceptual grouping, motion

## Abstract

Because object and self-motion are ubiquitous in natural viewing conditions, understanding how the human visual system achieves a relatively clear perception for moving objects is a fundamental problem in visual perception. Several studies have shown that the visible persistence of a briefly presented stationary stimulus is approximately 120 ms under normal viewing conditions. Based on this duration of visible persistence, we would expect moving objects to appear highly blurred. However, in human vision, objects in motion typically appear relatively sharp and clear. We suggest that clarity of form in dynamic viewing is achieved by a synergy between masking, perceptual grouping, and motion computation across retinotopic and non-retinotopic representations. We also argue that dissociations observed in masking are essential to create and maintain this synergy.

## Introduction

Studies at the turn of the 20th century analyzed the perception of moving form and laid foundations of important discoveries related to visual masking (e.g., McDougall, 1904; Piéron, 1935) as well as the relationship between form and motion processing (Kolers, 1972). Surprisingly, however, most of the studies during the last three decades have focused on static form perception, and very little is known about mechanisms underlying moving form perception. The goal of this paper is to provide a short overview of findings related to the perception of moving form and to lay the foundations of a theory of dynamic form perception. In this theory, masking, perceptual grouping, and motion computation interact within and across retinotopic and non-retinotopic representations of the stimuli.

**Figure 1.**
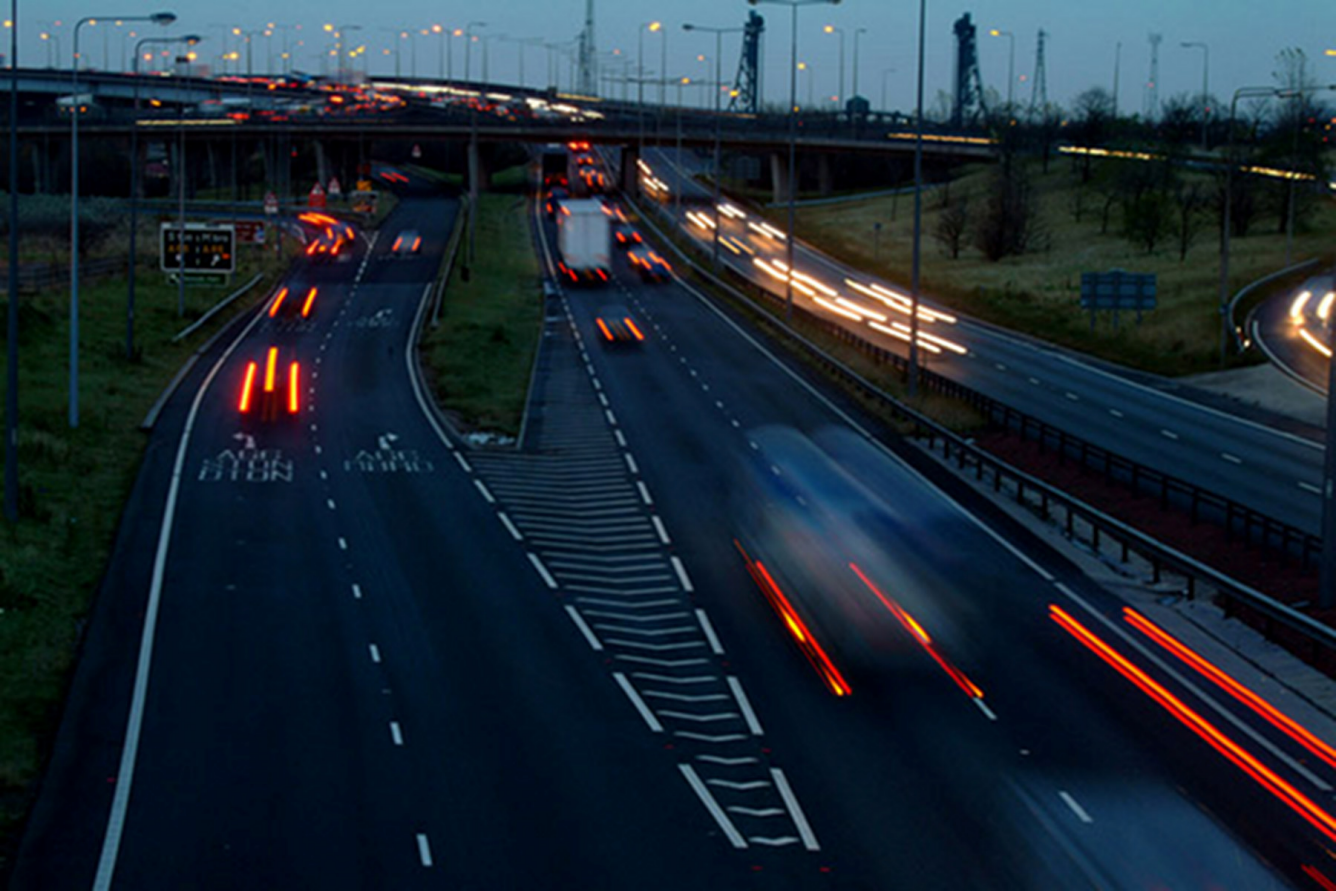
A picture taken at a shutter speed to illustrate the effect of visible persistence on blur. Reproduced with permission from FreeFoto.com.

The visible persistence of a briefly presented stationary stimulus is approximately 120 ms under normal viewing conditions (e.g., Haber & Standing, 1970; see also Coltheart, 1980). Based on this duration of visible persistence, one would expect moving objects to appear highly blurred. For example, a target moving at a speed of 10 deg/s should generate a comet-like trailing smear of 1.2 deg extent. The situation is similar to pictures of moving objects taken at an exposure duration that mimics visible persistence. As illustrated in Fig. 1, in such a picture, stationary objects are relatively clear but moving objects exhibit extensive blur.

Unlike photographic images, however, visual objects in motion typically appear relatively sharp and clear (e.g., Bex, Edgar, & Smith, 1995; Burr & Morgan, 1997; Farrell, Pavel, & Sperling, 1990; Hammett, 1997; Hogben & Di Lollo, 1985; Ramachandran, Rao, & Vidyasagar, 1974; Westerink & Teunissen, 1995). Because object and self-motion are ubiquitous in natural viewing conditions, understanding how the human visual system achieves a rela-tively clear perception for moving objects is a fundamental problem in visual perception. While pursuit eye movements can retinotopically stabilize a moving target and help reduce its perceived smear (Bedell & Lott, 1996; Tong, Patel, & Bedell, 2005), even under these conditions, the problem of smear remains for other ob-jects present in the scene. Furthermore, the initiation of an eye movement can take about 150–200 ms dur-ing which a moving object can generate considerable smear. In the next section we present evidence that one mechanism that contributes to the perceived clarity of moving objects is metacontrast masking. This is followed by a section that highlights the importance of dissociation properties of metacontrast in achieving this task. In the subsequent section, we argue that, while metacontrast masking can reduce the extent of smear for moving objects, the synthesis of form for moving objects necessitates non-retinotopic feature processing. This leads to the section where, we formulate specific hypotheses for dynamic form perception. Findings from anorthoscopic perception to provide empirical evidence for the proposed non-retinotopic form perception mechanisms are reviewed next. In the following section, we present our recent results indicating that non-retinotopic perception is not limited to anorthoscopic perception but applies to perception in general. Possible neural correlates for non-retinotopic mechanisms are discussed next. The final section concludes the manuscript.

## MOTION DEBLURRING IN HUMAN VISION

Burr (1980) and Hogben & Di Lollo (1985) measured the perceived extent of motion smear produced by a random array of moving dots as a function of exposure duration. For exposure durations shorter than approximately 40 ms, the extent of perceived smear increased with exposure duration, as one would expect from the visible persistence of static objects. However, for exposure durations longer than 40 ms, the length of perceived smear was much less than that predicted from the persistence of static targets. This reduction of perceived smear for moving objects has been termed “motion deblurring” (Burr, 1980; Burr & Morgan, 1997).

Contrary to the reports of motion deblurring, it has been long known that isolated targets in real motion (e.g., Bidwell, 1899; McDougall, 1904) and in apparent motion (Castet, 1994; Di Lollo & Hogben, 1985; Dixon & Hammond, 1972; Farrell, 1984; Farrell et al., 1990) exhibit extensive smear. In order to reconcile the apparently contradictory observations of motion deblurring for a field of moving dots and extensive smear for isolated moving targets, we conducted experiments in which the density of moving dots was varied systematically, ranging from a single dot to 7.5 dots/sq-deg (Chen, Bedell, & Öğmen, 1995). Our results showed that isolated targets moving on a uniform background are perceived with extensive motion blur and the reduction in the spatial extent of perceived motion blur (motion deblurring) increases as the density of moving dots in the array is increased. In other words, the motion deblurring reported by Burr (1980) is not a general phenomenon and applies principally to displays containing a relatively dense array of moving objects.

Several models have been proposed to explain motion deblurring based on a motion estimation procedure which is used to compensate for the adverse blurring effect resulting from the object motion (e.g. Anderson & van Essen, 1987; Burr, 1980; Burr, Ross & Morone, 1986; Martin & Marshall, 1993). According to Burr (1980) , motion estimation is achieved by the spatio-temporally oriented receptive fields of motion mecha-nisms. Martin and Marshall (1993) proposed a similar model wherein excitatory and inhibitory feedback connections suppress the persistent activity of neurons along the motion path. The “shifter-circuit” model of Anderson and van Essen (1987) uses an estimation of motion in order to generate a cortically localized (i.e. stabilized) representation of moving stimuli thereby avoiding the smear which would result from the change of cortical locus of neural activities. All these motion estimation/compensation models predict that an isolated moving target should produce no visual blur provided that it sufficiently stimulates the motion estimation/compensation mechanisms. However, as stated above, this prediction, is in sharp contradiction with the extensive blur observed for a moving isolated target (e.g. Bidwell, 1899; Chen et al., 1995; Lubimov & Logvinenko, 1993; McDougall, 1904; Smith, 1969a, b). In our study (Chen et al., 1995), by using several paradigms directly tailored to test the predictions of motion compensation models, we showed that the activation of motion mechanisms is not a sufficient condition for motion deblurring and that the reduction of perceived blur requires the presence of spatio-temporally adjacent targets. Taken together, these findings provide strong evidence against motion estimation/compensation models.

Several researchers suggested inhibition as a candidate mechanism for motion deblurring (e.g., Castet, 1994; Di Lollo & Hogben, 1985, 1987; Dixon & Hammond, 1972; Francis, Grossberg, & Mingolla, 1994; McDougall, 1904; Öğmen, 1993). Because inhibition is a rather general concept, it is important to determine how and where it operates to achieve motion deblurring. Empirical evidence supports the view that the inhibitory mechanisms underlying metacontrast masking are the ones involved in motion deblurring. Metacontrast masking refers to the reduced visibility of a target stimulus by a spatially non-overlapping and temporally following mask stimulus (Bachmann, 1984; Breitmeyer & Öğmen, 2000; 2006). Several studies using stimuli in apparent motion showed that the duration of visible persistence decreases as the spatial separation between successively presented targets is reduced (Castet, Lorenceau, & Bonnet, 1993; Di Lollo & Hogben, 1985; Farrell, 1984). Similarly, the metacontrast suppression of the target increases as the spatial separation between the target and mask decreases (e.g., Alpern, 1953; Breitmeyer & Horman, 1981; Growney, Weisstein, & Cox, 1977; Kolers & Rosner, 1960; Lefton, 1973). When the target and mask have similar energy, optimal metacontrast masking occurs when the mask follows the target approximately by 40–100 ms, depending on the stimulus parameters and task (rev. Breitmeyer & Öğmen, 2006). Breitmeyer and Horman (1981) showed that for high-contrast stimuli in apparent motion, optimal metacontrast occurred at a stimulus onset asynchrony of about 65–100 ms, depending on the spatial separation of the targets. Chen et al. (1995) reported that mo-tion deblurring is stronger in the periphery than in the fovea, in agreement with stronger metacontrast in the periphery in general (e.g., Alpern, 1953; Stewart & Purcell, 1974). Motion deblurring is closely related to “sequential masking” (Otto, Öğmen, & Herzog, 2006; Piéron, 1935) which in turn can be viewed as a form of metacontrast (Breitmeyer & Öğmen, 2006).

To test the relationship between metacontrast and motion deblurring computationally, we used a model of REtino-COrtical Dynamics (RECOD) (Öğmen, 1993), which has been applied to both paradigms. The general structure of this model is discussed in the next section. This model suggests that the main inhibitory process in metacontrast is the inhibition of sustained activities, originating from the parvocellular or P pathway, by transient activities, originating from magnocellular of M pathway (“transient-on-sustained inhibition”, see also Breitmeyer & Ganz, 1976). Simulations of the model for a widerange of metacontrast and motion deblurring data provided evidence that metacontrast masking is the key mechanism for motion deblurring (Breitmeyer & Öğmen, 2006; Purushothaman, Öğmen, Chen & Bedell, 1998). There is also clinical evidence supporting the model’s prediction that transient-on-sustained (M-on-P) inhibition plays a major role in motion deblurring: Tassinari et al. (Tassinari, Marzi, Lee, Di Lollo, & Campara, 1999) found that patients with a likely deficit in the M pathway, due to a compression of the ventral part of the pre-geniculate pathway, had substantially less motion deblurring than normal controls.

In summary: (1) isolated targets moving on a uniform background are perceived with extensive motion blur; (2) the presence of spatio-temporally proximal stimuli can reduce the spatial extent of perceived motion blur (motion deblurring); (3) motion mechanisms cannot account for motion deblurring; (4) metacontrast masking (theorized to occur as transient-on-sustained inhibition) can account for motion deblurring.

## DISSOCIATIONS IN METACONTRAST AND THEIR ROLE IN MOTION DEBLURRING

**Figure 2.**
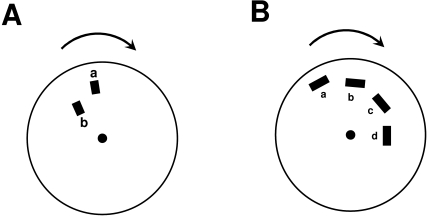
Stimulus arrangement used by A. McDougall (1904) and B. by Piéron (1935).

Figure 2 depicts the stimulus arrangements used by McDougall (1904) and Piéron (1935). McDougall reported that the blur generated by a leading stimulus (“a” in Fig. 2A) could be curtailed by adding a second stimulus (labeled “b” in Fig. 2A) in spatiotemporal proximity. This finding is in agreement with the more recent findings discussed in the previous section. Piéron (1935) modified McDougall’s stimulus to devise a “sequential” version as shown in Figure 2B. A notable aspect of the percept generated by this sequential version (see also Otto et al., 2006) is that, under appropriate parametric conditions, segment “a” can suppress the visibility of segment “b”, segment “b” in turn can suppress the visibility of segment “c”, etc. In other words, even though segment “b’’s visibility is suppressed, its effectiveness as a mask suppressing the visibility of segment “c” remains intact, i.e. a dissociation occurs between the visibility of a stimulus and its masking effectiveness.

Such a dissociation is necessary for metacontrast to act as an effective deblurring mechanism, otherwise motion blur would not be curtailed but transformed into an oscillatory profile. In the example of Figure 2B, without a dissociation between visibility and masking effectiveness, “b” would be invisible, but “c’ would be visible (because “b” would no longer be able to mask “c”) and this cycle of visibility and invisibility would repeat itself. The relationship between visibility and masking effectiveness in metacontrast was investigated systematically by Breitmeyer, Rudd and Dunn (1981). Their findings were modeled (Francis, 1997; Öğmen, Breitmeyer, & Bedell, 2006) and extended (Öğmen, Breitmeyer, Todd, & Mardon, 2006).

**Figure 3.**
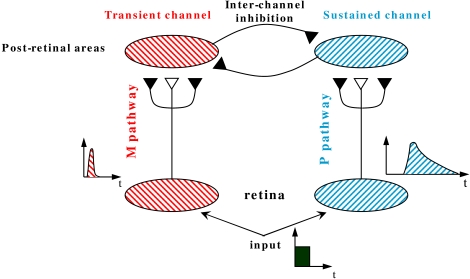
A schematic description of the RECOD model. The open and filled synaptic symbols depict excitatory and inhibitory connections, respectively. To avoid clutter, only a small part of the networks and connections are shown. The inter-channel inhibitory connection from the transient channel onto the sus-tained channel represents the interchannel "transient-on-sustained" inhibition.

Figure 3 provides a schematic description of the RECOD model (Breitmeyer & Öğmen, 2006; Öğmen, 1993) whose dual-channel structure can account for the dissociation between visibility and masking effectiveness. In this model, the input is conveyed to post-retinal networks through two major pathways corresponding to parvocellular (P) and magnocellular (M) pathways of the primate visual system. The post retinal areas receiving their major inputs from P and M pathways are also refered to as sustained and transient channels. In the model, the visibility of a stimulus as it relates to its brightness, contours, etc. is associated with activity in the sustained channels. The major suppressive effect in metacontrast is an inhibition from the transient channel on the sustained channel. Thus, because visibility and metacontrast masking effectiveness relate to two different processes, sustained and transient channel activities, respectively, the model can account for the aforementioned dissociation. The validity of this claim has been demonstrated by quantitative simulations (Öğmen, Breitmeyer, & Bedell, 2006; Öğmen, Breitmeyer, Todd et al., 2006). In summary, the RECOD model provides a mechanistic explanation of how motion deblurring can take place in retinotopic space.

The dual-channel structure of the model also allows it to account for another dissociation observed in visual masking (Öğmen et al., 2003): A U-shaped masking function can be obtained when observers make judgments related to the target’s surface (e.g., perceived brightness, contrast) and contour (e.g., contour completeness, contour shape) properties or figural identity (e.g., letter recognition). Under these conditions, if the observer’s task is changed to report the presence or the spatial location of the target, instead of its visibility, the metacontrast mask has no effect on the observer’s performance, as measured by simple/choice RTs or by response accuracy (e.g., Fehrer & Raab, 1962; Öğmen et al., 2003; Schiller & Smith, 1966). This dissociation can be readily explained by the RECOD model since target localization can be carried out by the activity in the transient channel ragardless whether the activity in the sustained channel is suppressed or not (for detailed predictions and a quantitative analysis, see Öğmen et al., 2003). This dissociation is important for the theory discussed in this manuscript in that, as we argue in the following sections, motion-induced perceptual grouping is essential for the computation of form for moving objects. The aforementioned dissociation suggests that transient, and by extension motion signals, remain intact under conditions in which the visibility of the stimulus is suppressed. As a result, motion-induced grouping operations can operate without being negatively affected by motion deblurring operations in the retinotopic space.

## FROM SHARPENED GHOSTS TO CLEAR FORMS: PROCESSING OF FORM INFORMATION FOR MOVING TARGETS OCCURS IN NON-RETINOTOPIC SPACE

Metacontrast mechanisms solve only partly the motion blur problem. If we consider the example shown in Fig. 1, metacontrast mechanisms would make the motion streaks appear shorter thereby reducing the amount of blur in the picture. Yet, although deblurred, moving objects would still suffer from having a ghost-like appearance. For example, in Fig. 1 notice the appearances of targets moving fast (e.g., the vehicles close to the observer), those that are moving more slowly (e.g., the white truck in the background approaching the traffic jam) and the stationary objects. The vehicles in front have a ghost-like appearance without any significant form information while the vehicles far, which move more slowly, have a more developed form, and finally static objects possess the clearest form. This is because static objects remain long enough on a fixed region of the film to expose sufficiently the chemicals while moving objects expose each part of the film only briefly thus failing to provide sufficient exposure to any specific part of the film. Similarly, in the retinotopic space, a moving object will stimulate each retinotopically localized receptive-field briefly and an incompletely processed form information would spread across the retinotopic space just like the ghost-like appearances in Fig. 1. We hypothesize that information about the form of moving targets is conveyed to a non-retinotopic space where it can accrue over time to allow neural processing to synthesize shape information.

## A THEORY OF MOVING FORM PROCESSING

We put forward the following hypotheses for the basis of moving form perception:

Hypothesis 1: Low-level encoding of moving stimuli occurs in a retinotopic space and metacontrast masking, theorized as transient-on-sustained inhibition, controls the extent of motion blur in this reti-notopic space.

Hypothesis 2: Accrual and processing of form information for moving objects occur in non-retinotopic space.

Hypothesis 3: The transfer of information from the retinotopic to the non-retinotopic space is guided by perceptual grouping operations.

Hypothesis 4: Non-retinotopic representation of moving objects consists of a joint representation of form and motion information. Motion vectors are specific to parts of objects.

Hypothesis 5: Phenomenal visibility of form requires correlated activity at both retinotopic and non-retinotopic spaces. Non-retinotopic activity that lacks correlated retinotopic activity leads to “dynamic amodal” perception (defined and discussed in the following section).

**Figure 4.**
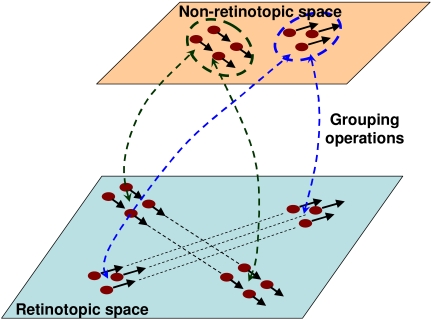
A schematic description of the proposed theory.

Fig. 4 provides a schematic description of the proposed scheme. In the retinotopic space, which is depicted at the bottom of the figure, two objects, one notional triangle composed of three dots and one notional square composed of four dots are shown moving in two different directions. Perceptual grouping operations determine, through space and time, the individual identities of objects. Observers perceptually group the dots into a triangular and a rectangular group based on the Gestalt principles of common fate (same velocity vector) and proximity. These perceptual grouping relations map in real-time the triangular and rectangular shapes to a non-retinotopic space where the accrual of information allows the processing of dynamic form perception. The accrual of information results from the fact that form information for a given object is mapped to the same group of neurons in the non-retinotopic space regardless the position of the object in the retinotopic space. Hence, these neurons can integrate and process this information over time. The dashed double-headed arrows between the retinotopic and non-retinotopic spaces indicate grouping-based mapping of activities. It is highly likely that grouping and form processing are interactive processes. The double-headed arrows in Fig. 4 are intended to depict such interactions. Notice that while the retinotopic position of the stimuli is changing in the retinotopic space, it remains fixed in the non-retinotopic space generating a “position-invariant” representation. This position-invariant representation allows the accrual, processing, and synthesis of form information for moving objects. It is likely that position-invariance involves multiple mechanisms. According to our model, perceptual grouping may play an important role in establishing and maintaining position-invariant representations. Assume that an object moves in the retinotopic space. When perceptual grouping identifies a stimulus configuration at some retinotopic neighborhood, *R*_0_ at time *t*_0_ to be the same object as a stimulus at a retinotopic location *R*_1_ at time *t*_1_, the corresponding activities are mapped to the same locus in the non-retinotopic space leading to a position-invariant representation. Perceptual grouping itself also involves several mechanisms and principles, “common fate” being one of them. Another example of motion-based grouping and non-retinotopic representation will be discussed in the section “Non-retinotopic perception is not restricted to anorthoscopic perception”.

The dashed ellipses around the objects in the non-retinotopic space highlight separate groups (objects). As depicted in the figure, form information in the non-retinotopic space is represented jointly with motion vectors. These motion vectors are associated with different parts of objects. Neurophysiologically, it is likely that these motion vectors are encoded in a separate area (e.g., MT+) and linked to the non-retinotopic space through grouping relations (see the Section “Potential neural correlates”). The dynamic grouping-based mapping between the two layers provides temporal correspondences between abstract form information in the non-retinotopic space and its underlying retinotopic activity. Our Hypothesis 5 states that phenomenal visibility at a given instant requires a correlated activity at both of these levels. This hypothesis is elaborated further in the next section where we apply the theory to anorthoscopic perception.

## ANORTHOSCOPIC PERCEPTION: A RETINOTOPIC IMAGE IS NOT NECESSARY FOR THE PERCEPTION OF FORM

**Figure 5.**
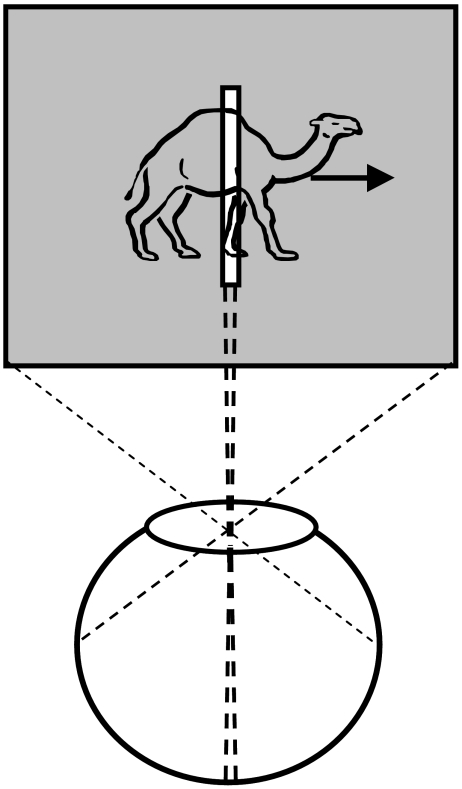
Depiction of the stimulus used in anorthoscopic perception experiments

The first part of the evidence to support our Hypothesis 2 (accrual and processing of form information for moving objects takes place in non-retinotopic space) comes from the classical phenomenon known as anorthoscopic perception (rev. Rock, 1981). The term anorthoscope refers to a device invented by Plateau in 19th century to demonstrate how static percepts can be generated from moving stimuli (Plateau, 1836). The anorthoscope consists of two disks rotating in opposite directions. One of the disks has slits through which parts of an image painted on the second disk are visible. In addition to leading to the development of contemporary cinematographic equipment, the anorthoscope also found use in scientific laboratories to study human perception (e.g., Helmholtz, 1867; Rothschild, 1922; Zöllner, 1862). The designs of this device and its contemporary computer emulations include a variety of versions depending on the number of slits, and on the combinations of whether the slit, the partially occluded image, and/or the eyes are moving (e.g., Anstis & Atkinson, 1967; Casco & Morgan, 1984; Fahle & Poggio, 1981; Haber & Nathanson, 1968; Mateeff, Popov, & Hohnsbein, 1993; Morgan, Findlay & Watt, 1982; Nishida, 2004). It is important to make distinctions between different configurations because they can activate drastically different visual mechanisms. Our main focus in this manuscript is for the case where there is only one stationary slit, the eyes are also stationary and an image moves behind the slit (Figure 5). Under these conditions, all information about the moving object’s shape collapses temporally on a narrow retinotopic locus in a fragmented manner, i.e. there is no spatially extended retinotopic image of the shape. Yet, observers perceive a spatially extended shape moving behind the slit instead of a fragmented pattern that is confined to the region of the slit. Thus, a retinotopic image is not necessary for the perception of form.

**Figure 6.**
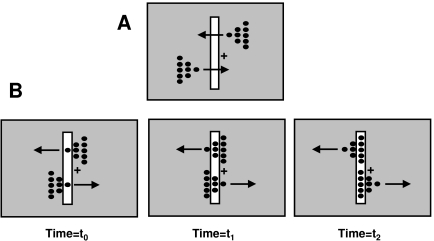
Stimulus configuration used to test retinal painting and time-of-arrival reconstruction theories.

**Figure 7.**
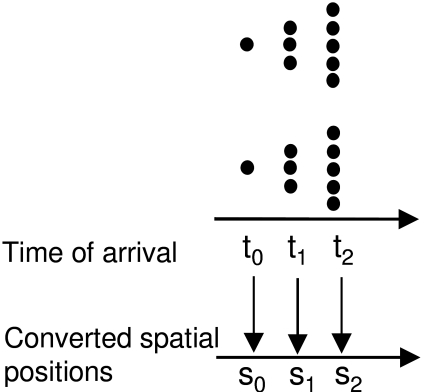
Prediction of the time-of-arrival reconstruction theory for the stimulus configuration shown in Fig. 6.

**Figure 8.**
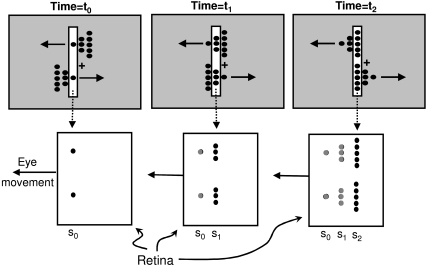
Prediction of the retinal painting theory for the stimulus configuration shown in Fig. 6.

The mechanisms underlying anorthoscopic perception are poorly understood. One of the early explanations, the “retinal painting” hypothesis (Helmholtz, 1867), was based on eye movements. If the eyes move while viewing the stimulus, then successive parts of the stimulus fall on adjacent retinotopic loci thereby “painting” a retinotopic picture of the figure. Subsequent research showed that while retinal painting can give rise to the perception of form, it cannot explain anorthoscopic perception in general: Measurement of eye movements and studies using retinal stabilization showed that anorthoscopic perception does occur in the absence of eye movements (Fendrich, Rieger, & Heinze, 2005; Morgan et al., 1982). The percepts resulting from eye movements can be explained simply by using the visible persistence characteristics of the human visual system. The critical findings to assess our theory are the ones in which an extended perception of the object occurs in the absence of eye movements. There have been two types of theories to explain anorthoscopic percepts in the absence of eye movements. According to Parks (1965), a post-retinal mechanism stores in memory the information available through the slit and reconstructs the figure according to a “time-of-arrival coding”. Figure 6 shows a stimulus configuration used to test both the retinal painting and the time-of-arrival reconstruction theories (McCloskey & Watkins, 1978; Sohmiya & Sohmiya, 1992, 1994). The stimulus consists of two triangular shapes moving in opposite directions. The tips of the triangles pass through the slit simultaneously, followed by the middle segments and finally the longest segments. Assume that the tip, the middle, and the base of the triangles cross the slit at *t*_0_, *t*_1_, and *t*_2_, respectively with *t*_0_<*t*_1_<*t*_2_. Observers are required to fixate on the fixation cross and report the perceived shape of stimuli. The time-of-arrival coding theory states that the time-of-arrival will be used to construct spatial form. As shown in Fig. 7, according to this theory these time-of-arrivals are converted to spatial positions *s*_0_, *s*_1_, and *s*_2_, respectively with *s*_0_<*s*_1_<*s*_2_. As a result, the theory predicts that the observers should perceive the two triangles pointing in the same direction. The same prediction is made by the retinal painting theory. This theory assumes that an involuntary eye movement shifts the retina with respect to the stimulus. Assume that the eye movement brings retinotopic positions *s*_0_, *s*_1_, and *s*_2_ in alignment with the slit at time instants *t*_0_, *t*_1_, and *t*_2_, respectively. As depicted in Fig. 8, this would result in the two triangles pointing in the same direction. However, observers’ perception corresponds to the actual stimulus configuration, i.e. the upper and the lower triangles pointing to the left and right, respectively (McCloskey & Watkins, 1978; Sohmiya & Sohmiya, 1992, 1994). Not only does this experiment reject these two theories but it also highlights an essential part of anorthoscopic perception: If the direction of motion is not known, the stimulus is ambiguous in that a leftward moving image and its mirror-symmetric version moving rightward generate identical patterns in the slit. Therefore, the determination of the direction of motion is critical for anorthoscopic perception. Indeed, anorthoscopic percepts consist of the shape moving in the correct direction. Our Hypothesis 4 incorporates this critical observation into our theory.

**Figure 9.**
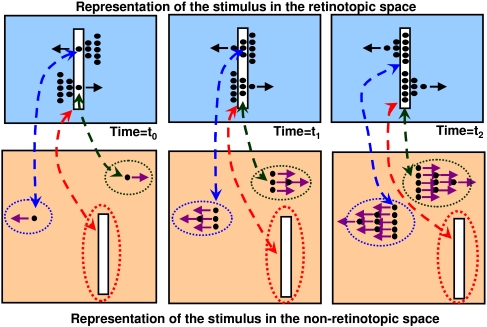
Prediction of the theory presented in this manuscript for the stimulus configuration shown in Fig. 6.

Fig. 9 depicts how our theory can account for the perception generated by the stimulus in Fig. 6. At time *t*_0_, the slit and the two dots constituting the tips of the triangles generate their representations in the non-retinotopic space with their corresponding motion vectors. For depiction purposes, each “object” in the non-retinotopic space is highlighted by a dashed ellipse. Note that because representation in the non-retinotopic space is position-invariant, the relative positions of different objects in this space do not carry spatial information. The relative spatial positions of different objects at a given time instant are encoded by their mapping via grouping operations to the retinotopic space (shown by the dashed lines with double-headed arrows between the retinotopic and non-retinotopic representations). At time *t*_1_ the array of middle dots are mapped according to grouping relations based on common fate and proximity so that the upper and lower dots map to the corresponding tip points. The relative positions of different parts of a given object in the non-retinotopic space are important, because they encode the shape of that object. We suggest that the motion direction vectors determine the relative position of the middle array with respect to the tip positions. For an object moving to the right (left), the temporally lagging part of the shape would be placed to the left (right) as is the case for the lower (upper) triangle. The same operation occurs at time *t*_2_ for the bases of the triangles. To complete the account of what is perceived, we need to consider the effects of occlusions.

**Figure 10.**
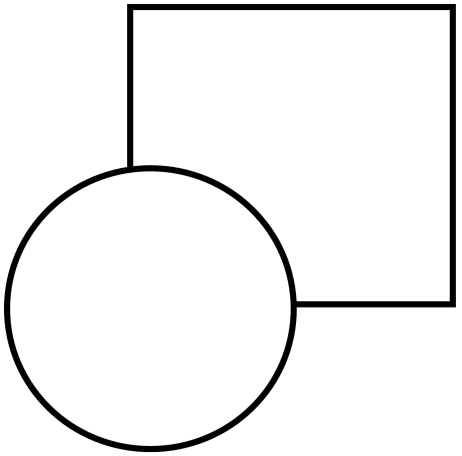
An example of a stimulus that leads to "amodal completion". Typically, observers perceive a square behind the circle, even though part of the square is not explicitly present in the image. This part is assumed to be present and occluded by the circle.

When viewing the stimulus shown in Fig. 10, observers typically “perceive” a circle and a square even though part of the square is not directly visible. This type of figural completion is called amodal completion (Michotte, Thinès, & Crabbé, 1964). From a terminological point of view, to distinguish this type of perception from the perception that arises in response to “directly visible” stimulus, we use the term *amodal* visibility as opposed to *phenomenal* visibility. What is perceived behind the slit in anorthoscopic perception can be viewed as a dynamic version of amodal visibility. Even though all parts of the figure passing behind the slit are not simultaneously visible, observers “perceive” the complete shape. For example, after the tip of the triangle falls behind the occluder, observers continue to perceive the tip moving forward even though they do not directly see it. To accommodate this amodal effect, we simply assume that, at any given instant, the retinotopic and non-retinotopic activities that are linked by perceptual grouping (e.g., the tips of the triangle for *t*_0_, the middle parts of the triangles for *t*_1_, etc. in Fig. 9) become phenomenally visible. At any instant, the activity in the non-retinotopic space that has no correlated activity in the retinotopic space would be perceived “amodally”. We designate this as *dynamic* amodal perception in that the non-retinotopic activity without correlated retinotopic activity will *appear to move* according to the velocity vector associated with that part of the figure.

Finally, let us point out that, due to the “aperture problem”, the recovery of motion and form information in anorthoscopic perception is illposed (e.g., Shimojo & Richards, 1986). Our theory relates shape and motion distortions reported in anorthoscopic percepts to the errors in estimation of velocity vectors.

## NON-RETINOTOPIC PERCEPTION IS NOT RESTRICTED TO ANORTHOSCOPIC PERCEPTION

 While anorthoscopic perception shows clearly that form perception can take place in the absence of a retinotopic image, generalization of underlying non-retinotopic mechanisms to normal viewing requires the demonstration of non-retinotopic perception without the use of occluders or slits. Previous research revealed illusions where features of objects are perceived non-retinotopically, i.e. at different locations than their retinotopic location. Treisman and Schmidt (1982) showed examples of illusory feature conjunctions when observers’ attention is divided. For example, in a small number of trials observers may report seeing a green square in response to a display containing red squares and green circles. This indicates that the retinotopic loci of the shape and color information can be incorrectly combined (Treisman & Schmidt, 1982). Because such illusory feature conjunctions typically occur when observers’ attention is divided, this illusion has been interpreted to reflect an error resulting from the limited attentional resources of the observer. 

**Figure 11.**
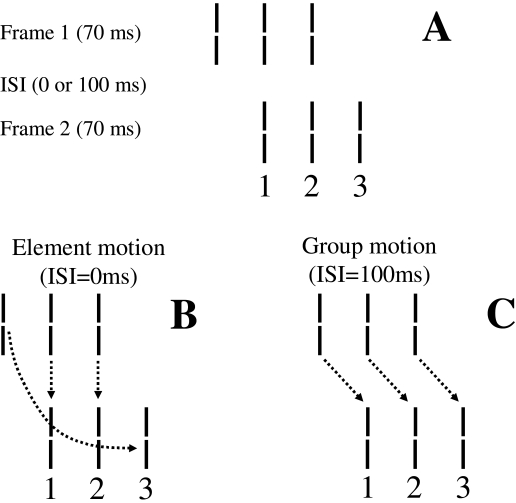
(A) A Ternus-Pikler display consisting of three lines. Correspondences in element (B) and group (C) motion percepts. From Öğmen et al. (2006c).

Similarly, many other feature mislocalizations in human vision have been attributed to “errors” stemming from limitations of perceptual processing such as masking (Stewart & Purcell, 1970; Stoper & Banffy, 1977; Werner, 1935; Wilson & Johnson, 1985), feature migration (Butler, Mewhort, & Browse, 1991; Herzog & Koch, 2001), feature misbinding in object substitution (Enns, 2002), crowding (Parkes, Lund, Angelucci, Solomon, & Morgan, 2001), pooling (Baldassi & Burr, 2000), sampling of continuous information stream (Cai & Schlag, 2001), distributed micro-consciousness (Zeki, 2001; Zeki & Bartels, 1998), and differential latencies (Arnold & Clifford, 2002; Bedell, Chung, Öğmen, & Patel, 2003). On the other hand, to provide support for our theory, we need to demonstrate cases of non-retinotopic perception that result not from errors of the visual system, but rather from its fundamental and lawful aspects. In particular, our Hypothesis 3 states that the transfer of information from the retinotopic to the non-retinotopic space is guided by perceptual grouping operations. Recently, by using a stimulus known as the “Ternus-Pikler display” (e.g., Dawson & Wright, 1994; Grossberg & Rudd, 1989; He & Ooi, 1999; Kramer & Yantis, 1997; Pantle & Picciano, 1976; Petersik, Schellinger, & Geiger, 2003; Petersik & Rice, 2006; Pikler, 1917; Ternus, 1926) we showed a new illusion where non-retinotopic feature perception obeys rules of perceptual grouping. Introduced by Gestalt psychologists, the basic Ternus-Pikler display consists of two frames separated by an inter-stimulus interval (*ISI*). The first frame contains a given number of elements (e.g., three line segments) and the second frame consists of a spatially shifted version of the elements of the first frame such that a subset of the elements spatially overlaps in the two frames. An example is shown in Fig. 11A where the two frames contain three elements arranged in such a way that two of the elements spatially overlap.

**Figure 12.**
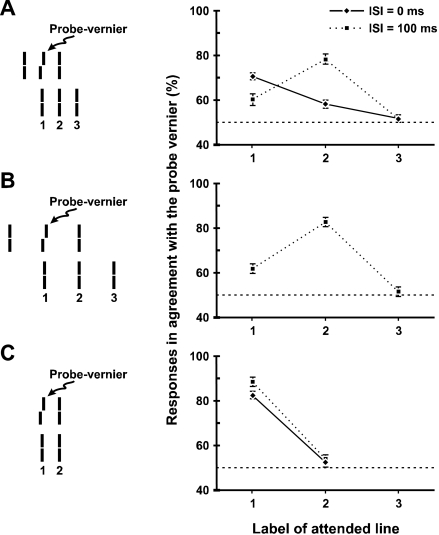
Experimental results for the Ternus-Pikler display with inter-element separation of 800 sec (A) and 1600 sec (B), as well as for the control condition (C) where no perception of motion is elicited. From Öğmen et al. (2006c).

These displays are designed to investigate factors that control how objects, or parts thereof, maintain their identities during motion. When *ISI* is short, the prevailing percept is that of *element motion* (Fig. 11B), i.e. the leftmost element in the first frame is seen to move directly to the rightmost element in the second frame while the two central elements are perceived stationary (as depicted by the dashed arrows in Fig. 11B). When *ISI* is long, the prevailing percept is that of *group motion*, i.e. the three elements in the first frame move as a single group to match the corresponding three elements in the second frame (as depicted by the dashed arrows in Fig. 11C). Thus the resulting percepts can be understood in terms of motion-induced grouping operations. In element motion, the leftmost element in the first frame and the rightmost element in the second frame are perceived as “one object” moving from left to right. The remaining two elements form together a second group. This latter two-element group is perceived stationary and matched with the two element group in the second frame according to the arrows in Fig. 11B. In group motion, the three elements in the first frame form a single group to match the corresponding elements of the three-element group in the second frame as shown by the arrows in Fig. 11C. Inserting a figural feature (a Vernier offset) to the central element in the first frame (“probe Vernier” in Fig. 12A) allowed us to investigate whether features are perceived according to retinotopic or according to perceptual grouping relations. Observers were instructed to attend to one of the Ternus-Pikler elements in the second frame, labeled as 1, 2, or 3 (see Fig. 12A) and to report the perceived direction of the Vernier offset (left or right) for this attended element. The offset direction for the probe Vernier was selected randomly in each trial. Naïve observers had no knowledge about where the Vernier offset was physically presented and no feed-back was given.

To derive specific predictions from retinotopic versus grouping-based hypotheses of feature attribution, consider first the retinotopic hypothesis. According to this hypothesis, features are perceived at the retinotopic positions where they are presented. Furthermore, features can be integrated retinotopically due to temporal integration properties of the visual system (Herzog, Parish, Koch, & Fahle, 2003). Consider for example the static control condition (Fig. 12C) which is identical to the Ternus display in Fig. 12A with the exception that the leftmost element of the first and the rightmost element of the second frame are omitted. In this control experiment no motion percept is elicited and the spatiotemporal integration combines the probe Vernier offset information retinotopically across the two frames. As shown in Fig. 12C, the percentage of responses in agreement with the probe Vernier is high for element 1 and near chance for element 2 for *ISI* = 0 and 100 ms. If the attribution of features in the two-frame display were made according to retinotopic relationships, we would expect a similar outcome for the Ternus-Pikler display provided that *ISI* is short enough to fall in the range where temporal integration occurs. Thus, we would expect the percentage of responses in agreement with the probe Vernier to be high for element 1 and near chance for elements 2 and 3 for *ISI* = 0 and 100 ms.

On the other hand, if attribution of features were made according to non-retinotopic relations, in particular according to motion-induced grouping, two different outcomes would be expected according to *ISI*: For short *ISI*s, because the central element in the first frame is perceptually identified with the element labeled 1 in the second frame (Fig. 11B), we would expect the percentage of responses in agreement with the probe Vernier to be high for element 1 as in the retinotopic case. At long *ISI*s, however, because the central element in the first frame is perceptually grouped with the element labeled 2 in the second frame (Fig. 11C), we would expect the percentage of responses in agreement with the probe Vernier to be high for element 2 even though there is no Vernier information at this retinotopic position.

**Figure 13.**
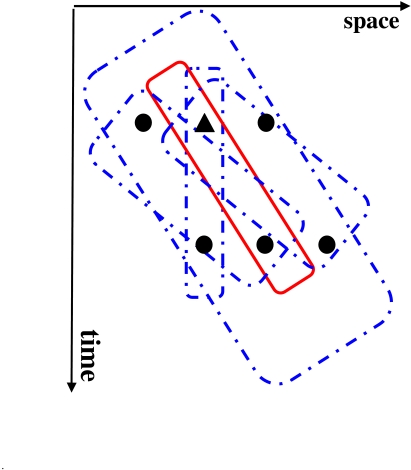
A space-time depiction of the Ternus-Pikler stimulus. For simplicity, one dimensional space is used and the offset and straight Verniers are indicated by triangle and circle symbols. A variety of spatio-temporally oriented receptive fields are superimposed on the stimulus. While the mechanism shown by solid red contour integrates Vernier information in accordance with the results shown in Fig. 11, the rest of the mechanisms, shown by dashed blue contours integrate in a way inconsistent with the data.

Results in Fig. 12A and B support the predictions of grouping based non-retinotopic feature perception hypothesis. Not only does this experiment [additional data in (Öğmen, Otto, & Herzog, 2006)] show non-retinotopic feature perception but it also highlights that grouping operations are critical in establishing the mappings from retinotopic to non-retinotopic space. A depiction of our stimulus in a space (horizontal axis) time (vertical axis) diagram is shown in Fig. 13. For simplicity a one-dimensional space is used. The circles and the triangle represent the spatial positions of the straight and offset (probe) Verniers, respectively. The Ternus-Pikler stimulus will activate a large number of integrative mechanisms, some of which are shown superimposed on the stimulus. To explain our data, only an exclusive subset of these mechanisms – specific to the spatial locus and to the prevailing grouping relation (shown by the solid line in the figure) can be in operation. The remaining mechanisms (shown by dashed lines) will integrate information in a manner inconsistent with our data. The oriented receptive-field and the shifter circuit models show two major deficiencies in explaining these data. First, because they do not take into account grouping mechanisms, they will integrate the Vernier information in multiple (inappropriate) ways following the activation of multiple motion detectors. Second, because they lack proper metacontrast mechanisms, they cannot predict when and how motion blur will be curtailed (Section “Motion deblurring in human vision”).

Pääkkönen and Morgan (1994) proposed a two-phase motion deblurring model wherein the first stage is “camera like exposure phase” that always produces motion blur. The second phase is proposed to carry out a “translation-invariant integration” of moving stimuli. This phase does not produce motion blur. No specific mechanisms were suggested for how translation-invariance is obtained. This model cannot explain the results discussed in the section “Motion deblurring in human vision”: Because motion blur in the first phase is assumed to be “camera-like”, the model predicts that motion blur should not to depend on the density of dots, contrary to the empirical findings. Neither can this model explain the results discussed in this section, because without grouping operations, the model cannot predict which specific translation will superimpose the elements in the two frames.

Our proposed theory goes beyond these previous models by including a retinotopic stage with “camera like” persistence *whose extent is controlled by metacontrast interactions*. Furthermore, the transition to non-retinotopic representation *is governed by perceptual grouping operations*, a property that allows us to explain Öğmen, Otto, & Herzog’s (2006) experimental results summarized in this section. The theory can also be applied to other non-retinotopic percepts observed in anorthoscopic viewing conditions.

## POTENTIAL NEURAL CORRELATES

The current neurophysiological knowledge of primate brain is not detailed enough to map directly our theory to neural structures. However, it is well known that early visual areas V1, V2, V3, V4/V8 and V3a are retinotopic and contain a complete eccentricity and polar angle map. Beyond retinotopic cortex, the polar angle representation becomes cruder. Interestingly, a recent study by Yin, Shimojo, Moore and Engel (2002) investigated neural correlates of anorthoscopic perception using fMRI. Their experiments included anorthoscopic percepts and control conditions with distorted stimuli that failed to generate anorthoscopic percepts. The activities in the retinotopic cortex did not correlate with whether the observers experienced anorthoscopic percepts or not. On the other hand, cortical activities in “object areas”, in the Lateral Occipital Complex (LOC), a mainly non-reti-notopic area, as well as those in the human motion area MT+ were correlated with anorthoscopic perception. Human motion area MT+, which is a likely homologue of the macaque motion-sensitive area MT/V5 (Heeger, Huk, Geisler, & Albrecht, 2000; Rees, Friston, & Koch, 2000), contains an orderly eccentricity organization within a hemifield representation (Dukelow, DeSouza, Culham, van den Berg, Menon & Vilis, 2001; Huk et al., 2002). LOC is a cortical region that exhibits selectivity to pictures of intact “meaningful” objects compared to scrambled objects and pictures that lack a clear meaningful object interpretation (Allison, Ginter, McCarthy, Nobre, Puce, Luby et al., 1994; Allison, Puce, Spencer & McCarthy, 1999; Doniger, Foxe, Murray, Higgins, Snodgrass & Schroeder, 2000; Faillenot, Toni, Decety, Gregoire & Jeannerod, 1997; Grill-Spector, Kushnir, Edelman, Itzchak & Malach, 1998; Grill-Spector, Kushnir, Hendler, Edelman, Itzchak & Malach, 1998; Grill-Spector, Kushnir, Hendler & Malach, 2000; Kanwisher, McDermott & Chun, 1997; Kourtzi & Kanwisher, 2000; Malach, Reppas, Benson, Kwong, Jiang, Kennedy et al., 1995; Murtha et al., 1999; Sergent, Ohta, & MacDonald, 1992). LOC also exhibits strong size and position invariance (Grill-Spector, Kushnir, Edelman, Avidan-Carmet, Itzchak & Malach, 1999; Malach et al., 1995). Hence, LOC and other similar non-retinotopic areas showing object selectivity can be candidates for our “non-retinotopic space”. Yin et al.’s (2002) study suggests that the motion vectors, directly depicted in the non-retinotopic area in Fig. 4, may physically reside in area MT+. A recent study by Kim & Kim (2005) provides evidence that LOC has direct connections to MT+ and V3A and that MT+ and V3A have reciprocal connections. V3A is part of the V3 complex which has been implicated in the analysis of dynamic form (Zeki, 1991). Thus a tentative mapping would include areas extending to V3 complex as our retinotopic space, LOC as the non-retinotopic space, and the connectivities between MT+, V3A, and LOC establishing the coupling of dynamic form and motion vector representations between these areas. While this mapping is highly speculative at this point, we believe that future neurophysiological studies can test more directly neural correlates of the proposed functional theory.

## CONCLUDING REMARKS

The three-dimensional structure of an object is mapped through the optics of the eye on two-dimensional retinae creating a “retinotopic image” of the object. Retino-cortical pathways provide an orderly projection to the lateral geniculate nucleus and to the primary visual cortex so that neighboring points on the retina map to neighboring points in these areas, a property known as retinotopy. This retinotopic organization is found in numerous visual cortical areas. Through their “classical” receptive fields, neurons in these visual areas process information locally in the retinotopic space. Retinotopic organization and retinotopically localized receptive-fields have been two fundamental pillars upon which most theoretical accounts of visual form perception are built. However, these theories are based mainly on a static characterization of visual perception and focus on how form information is processed for static objects. On the other hand, very little is known on how the nervous system computes the form of moving objects. Based on an analysis of dynamic aspects of vision, we argued that non-retinotopic computational principles and mechanisms are needed to compute the form of moving objects. We designate as “non-retinotopic” those mechanisms that can generate perception of form in the absence of a retinotopic image. Indeed, perceptual data demonstrate that a retinotopic image is neither necessary nor sufficient for the perception of form: When a moving object is viewed behind a narrow slit cut out of an opaque surface (anorthoscopic perception, Fig. 5), all information about the moving object’s shape collapses temporally on a narrow retinotopic locus in a fragmented manner, i.e. there is no spatially extended retinotopic image of the shape. Yet, observers perceive a spatially extended and perceptually integrated shape moving behind the slit instead of a series of fragmented patterns that is confined to the region of the slit. Anorthoscopic perception shows that a retinotopic image is not necessary for the perception of form.

The visibility of a “target stimulus” can be completely suppressed by a retinotopically non-overlapping “mask stimulus” that is presented in the spatio-temporal vicinity of the target stimulus, phenomena known as para- and metacontrast masking (Bachmann, 1984; Breitmeyer & Öğmen, 2006). These masking effects indicate that the existence of a retinotopic image is not a sufficient condition for the perception of form and that the dynamic context within which the stimulus is embedded plays a major role in determining whether form perception will take place.

In this manuscript, we presented a theory of moving form perception where masking, perceptual grouping, and motion computation interact across retinotopic and non-retinotopic representations. Due to visible persistence, moving targets are expected to generate extensive blur in retinotopic representations implemented in early visual cortex. We provided evidence showing that metacontrast masking controls the spatial extent of this blur. While this first step is critical in limiting the deleterious effect of motion blur; the computation of clear percepts for moving objects requires a non-retinotopic representation where figural information about moving objects is processed. We argued that motion-induced grouping is critical in transferring information from the retinotopic to non-retinotopic space. Dissociation between visibility and masking effectiveness allows metacontrast to be effective in a sequential mode. The RECOD model captures this property. The RECOD model can also explain the dissociation between visibility and spatial localization. This dissociation, allows the computation of motion information that can lead to motion grouping under metacontrast suppression conditions. Thus, taken together RECOD can implement the deblurring of retinotopic activity while preserving information for motion-induced grouping. In addition to normal viewing conditions, the proposed theory can also be applied to anorthoscopic perception which provides strong evidence that a “retinotopic image” is not necessary for the synthesis of a spatially extended percept. Our current work focuses on the interactions between perceptual grouping operations and non-retinotopic representations in order to develop a more detailed quantitative account for the remaining parts of the theory.
